# coreMRI: A high-performance, publicly available MR simulation platform on the cloud

**DOI:** 10.1371/journal.pone.0216594

**Published:** 2019-05-17

**Authors:** Christos G. Xanthis, Anthony H. Aletras

**Affiliations:** 1 Department of Clinical Sciences Lund, Clinical Physiology, Skåne University Hospital, Lund University, Lund, Sweden; 2 Laboratory of Computing, Medical Informatics and Biomedical–Imaging Technologies, School of Medicine, Aristotle University of Thessaloniki, Thessaloniki, Greece; McLean Hospital, UNITED STATES

## Abstract

**Introduction:**

A **C**loud-**OR**iented **E**ngine for advanced **MRI** simulations (coreMRI) is presented in this study. The aim was to develop the first advanced MR simulation platform delivered as a web service through an on-demand, scalable cloud-based and GPU-based infrastructure. We hypothesized that such an online MR simulation platform could be utilized as a virtual MRI scanner but also as a cloud-based, high-performance engine for advanced MR simulations in simulation-based quantitative MR (qMR) methods.

**Methods and results:**

The simulation framework of coreMRI was based on the solution of the Bloch equations and utilized a ground-up-approach design based on the principles already published in the literature. The development of a front-end environment allowed the connection of the end-users to the GPU-equipped instances on the cloud. The coreMRI simulation platform was based on a modular design where individual modules (such as the Gadgetron reconstruction framework and a newly developed Pulse Sequence Designer) could be inserted in the main simulation framework. Different types and sources of pulse sequences and anatomical models were utilized in this study revealing the flexibility that the coreMRI simulation platform offers to the users. The performance and scalability of coreMRI were also examined on multi-GPU configurations on the cloud, showing that a multi-GPU computer on the cloud equipped with a newer generation of GPU cards could significantly mitigate the prolonged execution times that accompany more realistic MRI and qMR simulations.

**Conclusions:**

coreMRI is available to the entire MR community, whereas its high performance and scalability allow its users to configure advanced MRI experiments without the constraints imposed by experimentation in a true MRI scanner (such as time constraint and limited availability of MR scanners), without upfront investment for purchasing advanced computer systems and without any user expertise on computer programming or MR physics. coreMRI is available to the users through the webpage https://www.coreMRI.org.

## 1 Introduction

Magnetic Resonance Imaging (MRI) simulations have been utilized to date mostly as a research tool to provide insight with respect to pulse sequence design [1–3] and as hardware development/improvement [4] as well as an educational platform to provide basic understanding of the MRI physics to students, physicians and technologists [5–7]. However, due to their rather limited scope and in order to reduce the complexity of the magnetic resonance (MR) experiment so as to execute the simulation within reasonable times, these MR simulators made several assumptions with respect to the underlying MR physics framework which limit their application in more realistic simulations.

In recent years, more advanced and general-purpose MR simulation platforms have been presented in the literature [8, 9]. SIMRI [8] was mainly developed for training purposes and consisted of a Graphical User Interface (GUI) for manipulating a number of MR protocol parameters whereas JEMRIS [9] provided a comprehensive, open-source solution for answering more complex questions on pulse sequence design and its performance was evaluated on a 32-dual-core CPU cluster. Although these simulation platforms allowed for application in large-scale analysis, they required advanced technical knowledge, advanced setup of a computer cluster and the ability to modify the source code. More recently, the latest advancements in graphics processing unit (GPU) technology and general-purpose computing on graphics processing units (GPGPU computing) allowed for complex large-scale MR simulations on a single computer without requiring simplifications of the MRI physics model so as to yield reasonable execution times [10–13]. MRISIMUL [12, 13] was the first comprehensive MR simulation platform that utilized GPU-technology and brought the computational supercomputer-level power or a computer cluster on a single, GPU-equipped, personal computer whereas MRiLab [11] was an open-source MRI simulation tool that utilized GPU-technology and allowed for a flexible representation of tissues by multi-pool exchange models. However, despite the latest progress on GPU-based MR simulations, the utilization of GPU-based MR simulation platforms remained limited since it required the purchase of an expensive computer system and advanced programming and installation expertise. Furthermore, the increased spatial resolution provided by newer MR systems and the incorporation of more realistic aspects in simulations (such as motion) may require the utilization of more advanced computer systems [12] equipped with the latest GPU technology available. In addition to this, research groups around the world have shown during the last few years an increasing interest in quantitative MRI by means of advanced MR simulations. Novel techniques, such as Magnetic Resonance Fingerprinting [14] and parallel Simulations for QUantifying RElaxation Magnetic Resonance constants (SQUAREMR) [15], utilize MR simulations to significantly improve quantitative MRI through the generation of large databases of simulated MR signals. Recently, Kantasis et al. [16] demonstrated how SQUAREMR performance could be improved through a cloud-based implementation that distributes the computational load to several GPU-enabled nodes and through the optimization of the parameter space that reduces the computational load without compromising the quantitative estimates. However, this solution was limited to SQUAREMR applications, was not accessible by the users and required the setup of an advanced computer architecture on the cloud. Last but not least, there is no simulation standard today within the MR field. A variety of MR simulators have been published in the literature to date, but they have mainly been developed and utilized for specific MR applications and they have not been validated extensively over a wider range of MR applications [17, 18].

All of the above point out the need for having an advanced MR simulation platform as a service, which can be easily accessible by the entire MR community, will remain common across research groups and will not require programming expertise. This simulation platform will allow researchers, technologists, physicians and students to design and configure advanced MR experiments without the time constraint imposed by costly experimentation on an MRI scanner or by the limited availability of MR scanners for research purposes. In particular, such a simulation platform could be used in MR research for answering particular methodological problems, providing insight with respect to future pulse sequence development, improving the idea-to-product time and reducing the need for MR experiments on human volunteers, patients and animals. In the field of MR education, such a simulation platform could be used for improving the way MR education is performed today by generating educational material that cover a wide range of variations of both the imaging protocol and the computer model, which is not easy today with a true MRI experiment configuration (MRI system, patient recruitment, etc.). Moreover, such a simulation platform could offer a hands-on experience to students, physicists, technologists and physicians that would like to investigate how changes on the configuration of an MR experiment affect the quality of the final MR image.

The specific aim of this study was the development of an advanced MR simulation platform (**C**loud **OR**iented **E**ngine for advanced **MRI** simulations—coreMRI) delivered as a web service through an on-demand, scalable cloud-based and GPU-based infrastructure. We hypothesized that such an online MR simulation platform could be utilized 1) as a virtual MRI scanner delivered worldwide through the cloud that simulates the entire MRI process, from pulse sequence development to signal generation and image formation, and 2) as a cloud-based, high-performance engine for advanced MR simulations in simulation-based quantitative MR methods [14, 15].

## 2 Materials and methods

**Fig 1:**
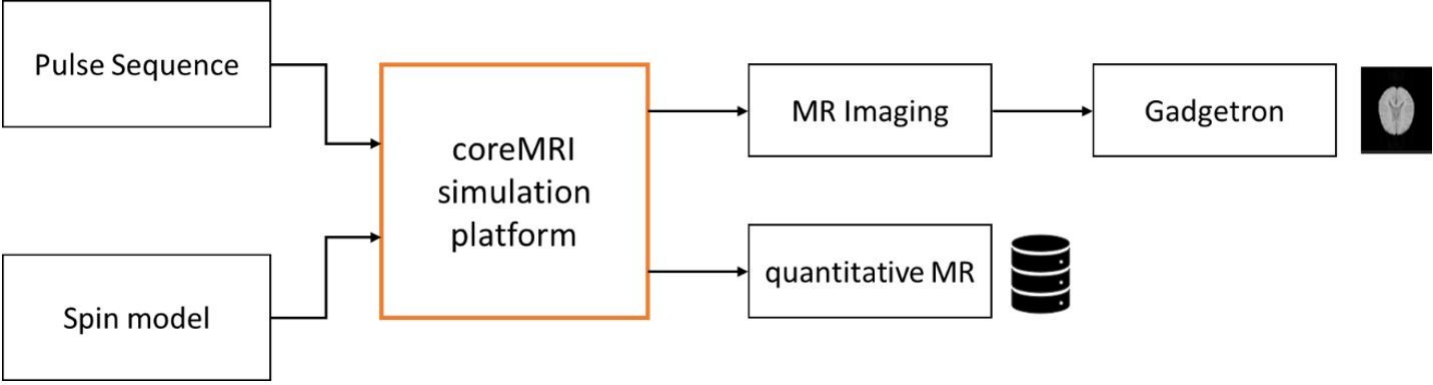
Simplified block diagram of the coreMRI simulation platform. The computational kernel (in bold orange) accepts two inputs: the pulse sequence and the spin model. Depending on the mode (MR Imaging or quantitative MR), the output of the coreMRI simulation platform is either raw simulated MR data that can be reconstructed (optionally) to a simulated MR image or a database of simulated MR signals. The modular design of the coreMRI simulation platform modular allows individual modules (such as the Gadgetron reconstruction framework [19]) to be added in the simulation workflow.

A simplified block diagram of the coreMRI simulation platform is shown in Fig 1. The entire solution was installed on the cloud and is reachable from any web browser through the domain name www.coremri.org. A Secure Sockets Layer (SSL) was established to provide an encrypted connection between the web browser and the web server on the cloud.

The simulation framework of coreMRI utilized a ground-up-approach design based on the principles already published in the literature [12, 13]. The coreMRI simulation platform of the Bloch equations was based on a modular design where individual modules (such as the Gadgetron reconstruction framework [19]) could be inserted in the main simulation framework. Amazon Web Services (AWS—aws.amazon.com) were utilized for the provision of cloud-based and GPU-based services to the end-users. The development of a web-server (front-end) allowed the connection of the end-users to the GPU-equipped instances (back-end) on the cloud.

### 2.1 coreMRI platform architecture: Front-end system

The front-end system was mainly employed as a job-manager that 1) activated the GPU resources required for the MR simulations, 2) distributed the data to the GPU instances, 3) collected the data generated from the GPU instances and 4) delivered the MR simulation data to the end-users. The front-end system is accessible to the end-users through the web (www.coremri.org) and consists of a web-page and a web-application. The web-page was built on a Joomla framework and acted as a user and content management system. The web-application was built with the use of HTML5, CSS3, bootstrap 3, JavaScript, jQuery, Java (as a middleware) and MySQL. The web-application was the main simulation platform of coreMRI that bridged the end-user with the cloud-based and GPU-based infrastructure, prepared the MR simulations, activated the GPU-resources required for the MR simulations and presented the simulated MR data. Once the simulation is ordered, the web-application requests through the AWS SDK (Software Development Kit) for PHP the initiation on the cloud of the type of the GPU-equipped instance that the user selected. A more detailed description of this process is given in section 2.2.

**Fig 2:**
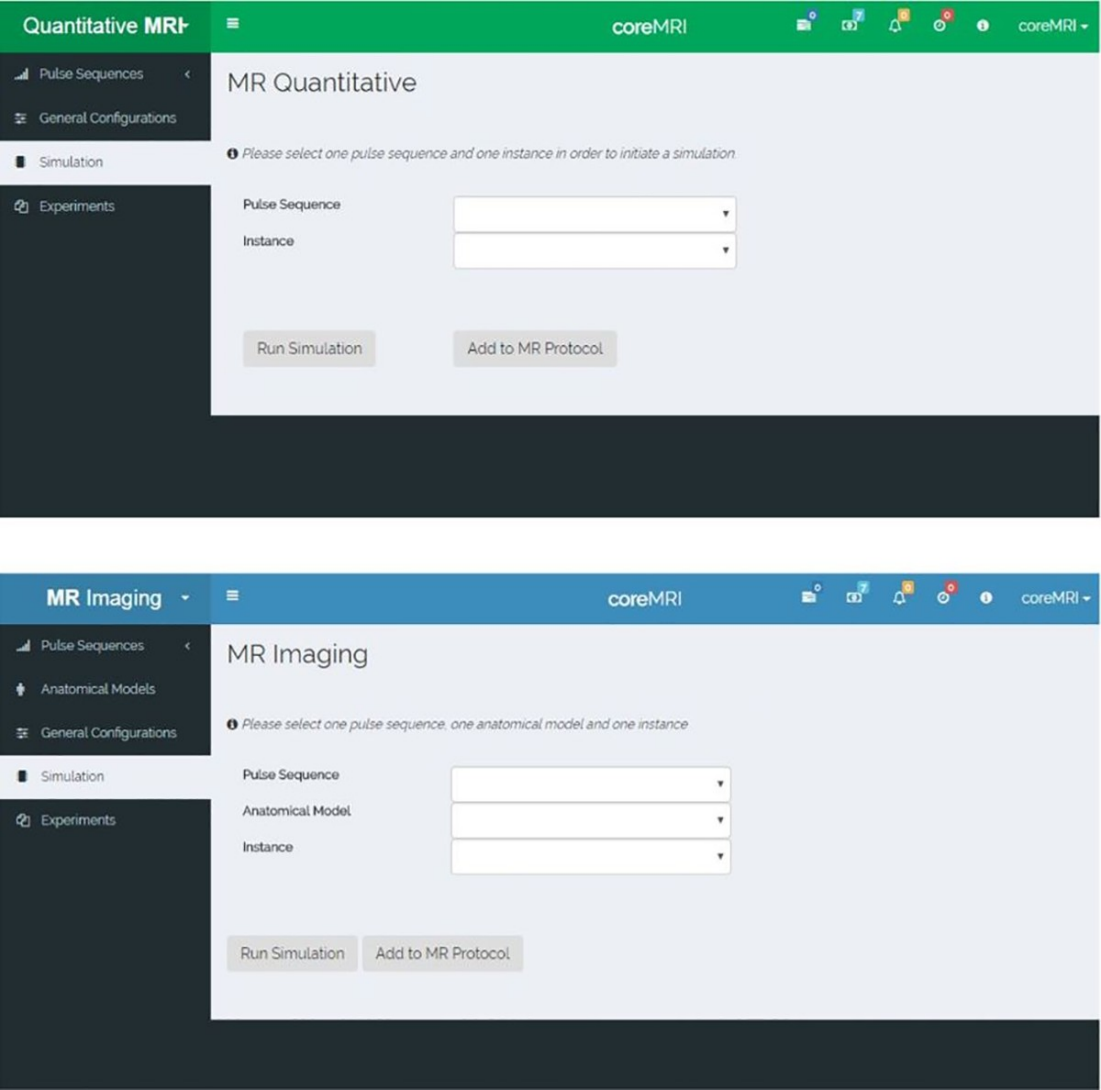
Screenshots of the web-application of the coreMRI simulation platform and the two simulation modes. A) The MR imaging mode simulates the entire MR process and colors the web-application in blue. B) The quantitative MR mode utilizes MR simulations for the generation of databases of simulated MR signals and colors the web-application in green.

Fig 2 shows screenshots of the web-application. The coreMRI simulation platform is available in two different modes: Magnetic Resonance Imaging (MR Imaging) and quantitative Magnetic Resonance (quantitative MR). The MR Imaging mode simulates the entire MRI process, from pulse sequence development and selection of the anatomical model to signal generation and image formation. The quantitative MR mode utilizes MR simulations for the generation of databases of simulated MR signals to be used in simulation-based quantitative MR methods, such as MRF and SQUAREMR [14, 15]. The MR Imaging mode colors the web-application in blue (Fig 2A), whereas the quantitative MR mode colors the web-application in green (Fig 2B). The user can switch between the two modes by clicking on the top left corner of the coreMRI simulation platform. Last, at the top-right corner of the web-application there is an area with icons that provide important information to the user, such as the remaining GPU-hours that the user has available for MR simulations, notifications on the status of the MR simulations, the number and type of GPU instances the user is currently using, the version of the coreMRI platform, etc.

#### 2.1.1Magnetic Resonance (MR) imaging mode

The MR Imaging mode requires the selection of one pulse sequence and one computer model for the execution of the MR simulation and the presentation of the outcome.

In more detail, the list with the pulse sequences is available through the tab “Pulse Sequences” from the left-side menu (Fig 2A). In this page, the user is allowed to 1) upload pulse sequences in a .mat (MATLAB) file format, 2) view/download the uploaded pulse sequences, 3) share pulse sequences with the coreMRI community, 4) view the pulse sequences that other users have uploaded and shared (through the Public Repository), and 5) design new pulse sequences through an online pulse sequence designer. More details about the pulse sequence designer are given in a separate section (Section 2.1.3).

The selection of the anatomical model is performed through the tab “Anatomical Models” from the left-side menu (Fig 2A). In this page, the user is allowed to 1) upload anatomical models in a .mat file format, 2) view/download the uploaded anatomical models, 3) share anatomical models with the coreMRI community, 4) view the anatomical models that other users have uploaded and shared (through the Public Repository).

The tab “Simulation” from the left-side menu (Fig 2A) allows the user to select the pulse sequence, the anatomical model, and the type of the GPU-equipped instance on the cloud that will perform the MR simulation. On this page, the user can also define/edit more characteristics of the MR simulation, such as the gyromagnetic ratio, the motion of the anatomical model, the reconstruction method, the magnetization transfer (MT) effect, etc.

The structure of the anatomical model allowed for a more complex tissue configuration. The basic tissue configuration involved the assignment of every single isochromat in 3D space with a set of basic MR characteristics (such as T1, T2 and proton density). The user could optionally assign more complex characteristics at every single isochromat in 3D space, such as chemical shift and parameters that define a two-pool model of MT exchange [20] that consists of a liquid pool (water protons) and a small semisolid pool (macromolecular content). Moreover, the structure of the anatomical model allowed for the utilization of multiple isochromats at the same location. Once the anatomical model was structured with more complex characteristics, their activation was performed through the proper selection of characteristics of the MR simulation in tab “Simulation”, such as activation of the MT module.

The tab “Experiments” from the left-side menu (Fig 2A) presents the current status for all the MR simulations performed by the user along with the MR simulation output for all the MR simulations that completed successfully. In this page, the user is allowed to 1) download the raw data of the MR simulation in a .mat file format, 2) view and download the Gadgetron-reconstructed images for the MR simulations if this option had been selected, and 3) view the log and characteristics of each MR simulation.

#### 2.1.2 Quantitative Magnetic Resonance (MR) mode

The quantitative MR mode requires the selection of one pulse sequence and the definition of the size of the spin model and range of spin parameters for the execution of the MR simulation and the presentation of the outcome.

In more detail, the list with the pulse sequences is available through the tab “Pulse Sequences” from the left-side menu (Fig 2B) in a similar manner as described in section 2.1.1. It should be noted, though, that the list of pulse sequences in the quantitative MR mode presents different pulse sequences from the ones presented in the MR imaging mode.

The tab “Simulation” from the left-side menu (Fig 2B) allows the user to select the pulse sequence and the type of the GPU-equipped instance on the cloud that will perform the MR simulation. On this page, the user also defines the characteristics of the spin model such as the range of the T1 and T2 values of the spin population, the position of the spins along the slice direction, the main field inhomogeneity that each spin experiences, etc. More characteristics of the MR experiment can also be defined/edited on this page, such as the gyromagnetic ratio, the magnetization transfer effect, etc.

The tab “Experiments” from the left-side menu (Fig 2B) presents the current status for all the MR simulations performed by the user along with the MR simulation output for all the MR simulations that completed successfully. On this page, the user is allowed to download the database with the simulated MR signals in a .mat file format and view the log and characteristics of each MR simulation.

#### 2.1.3 Pulse sequence designer

**Fig 3:**
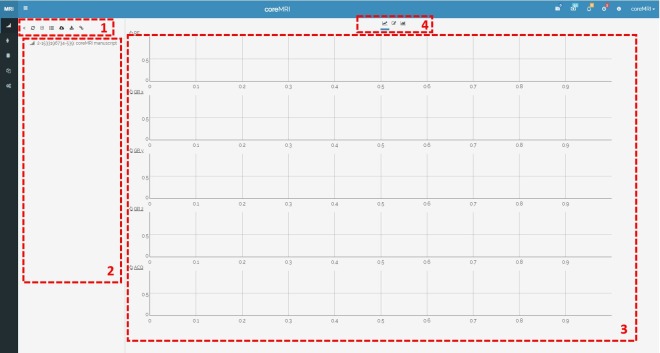
Layout of the pulse sequence designer. The layout involves four different areas: 1) the action buttons, 2) the tree-view representation of the pulse sequence, 3) the graphical representation of the pulse sequence, and 4) the tab area.

A Graphical User Interface for pulse sequence design was developed using HTML5, CSS3, JavaScript and MySQL. The Pulse Sequence Designer is available through the coreMRI framework, and its layout is shown in Fig 3.

Pulse sequences are designed in the tree-view interface and the sequence events are organized in blocks of RF, gradient and acquisition objects. Each block holds up to one object of each category (RF pulse, acquisition object, gradient pulse on X axis, gradient pulse on Y axis and gradient pulse on Z axis), whereas custom-designed RF and gradient pulses can be imported into the Designer. In addition to the regular types of objects, artificial types of objects (such as software crushers and single acquisition objects) can also be introduced in the design of the pulse sequence. Software crushers [12] is a form of crushers that apply on single time-points of the pulse sequence and induce instant nullification of the transverse components of all isochromats within the anatomical object. The single acquisition objects apply also to single time-points of the pulse sequence and define when the MR simulator records the transverse components of every spin of the spin model in simulation-based quantitative MR methods, such as SQUAREMR. The number of the single acquisition objects in the pulse sequence and the number of spins in the spin model define the size of the database of the simulated MR signals in such methods.

Each object of the pulse sequence designer is assigned with a set of attributes, whereas functional programming can be applied for calculating the parameters dynamically. The blocks can be further grouped in groups of blocks, whereas copy-paste actions allow for easy deployment of long pulse sequences. The group of blocks, the blocks and the objects that belong to the same category (such as RF pulses, gradient pulses, etc.) receive a unique increasing ID to facilitate the functional programming. The operation of functional programming allows the formation of mathematical expressions with 1) the global parameters, 2) the attributes of the objects that belong to the same block, and 3) the attributes of the parent block and group of blocks. Moreover, the graphical representation of the objects on the right side of the designer allows the users to view the changes performed on the individual objects within the selected block.

The pulse sequences developed in the designer can be exported in a format compatible either with the coreMRI simulation platform (.mat) or with the pulseq (.seq) framework [21]. Pulse sequences in pulseq (.seq) format can also be loaded in the designer either for visualization purposes or for further processing. The combination of the Sequence Designer with the pulseq framework allows the execution of the .seq files on real MR scanner with the use of hardware-dependent interpreters and their direct comparison with their simulation on the coreMRI simulation platform. Last, the designer allows the user to define an ISMRMRD header [22] that accompanies the pulse sequence throughout the simulation process and facilitates the reconstruction of the simulated MR data on the Gadgetron.

### 2.2 coreMRI platform architecture: Back-end system

The back-end system consists of instances on the cloud equipped with GPU cards that run the computationally demanding core services of the MR simulations. An Amazon Machine Image (AMI) of an Ubuntu 14.04 LTS instance was created and was used to launch new instances on the cloud. The NVIDIA drivers, the CUDA toolkit and the MATLAB Compiler Runtime (MCR) along with the Docker Engine and the Docker image of Gadgetron were included in the AMI.

Once the user defines the pulse sequence, the spin model, the total number and the type of the available GPU cards for the MR simulation and initiates the MR simulation in the front-end web-application, the front-end system uses the AMI to launch in the back-end a new instance with the GPU characteristics (GPU model and number of GPU cards) requested by the user. Once the GPU-equipped instance initiates, the components of the MR simulation (pulse sequence, anatomical model and configuration parameters) are transferred from the front-end system to the back-end system through a scalable file system. The MR simulation is performed through a compiled MATLAB application based on the mathematical model that describes the MR physics and has already been introduced in the literature [12, 13]. Distribution of data is performed through the MATLAB single-program-multiple-data (spmd) framework, whereas CUDA-C is utilized for the computationally intensive simulations on the GPUs (kernel). The anatomical model is divided evenly in space based on the available GPU cards and the characteristics of the GPU card, such as number of multi-processors, global memory, shared memory, registers, etc. and the kernel is called for every part of the anatomical model. The simulation computational kernel executes the matrix operations for every isochromat and for every time-step of the pulse sequence. These operations involve the calculation of the isochromat’s magnetization vector and the position (when motion models are included in the simulation). For every time-step of the pulse sequence that belongs to the acquisition window, the components of the net magnetization vector of the corresponding part of the anatomical model are stored in the global memory of the GPU. At the end of every kernel call, these data are transferred to the host CPU and the k-space matrix of the object is stored. At this stage, noise is added to the simulated signal (optional). The noise is in the form of an array of random floating-point numbers that are drawn from a normal distribution with zero mean and user-defined standard deviation. At the end of the simulation, the raw data are directed to the Gadgetron system for reconstruction purposes (if applicable) and then they are transferred to the front-end system through the scalable file system.

For this study, Amazon g2-type (up to 4 GPUs, NVIDIA GRID K520, 1536 CUDA cores and 4GB GPU memory), g3-type (up to 4 GPUs, NVIDIA Tesla M60, 2048 CUDA cores and 8GB GPU memory) and p2-type (up to 16 GPUs, NVIDIA K80, 2496 CUDA cores and 12GB GPU memory) instances were utilized.

A full documentation of the coreMRI simulation platform along with the latest updates and the format of all the .mat files can be found in www.coremri.org/documentation.

### 2.3 Simulated experiments

#### 2.3.1 MR imaging mode: Pulse sequence designer

Three Gradient Recalled Echo (GRE) pulse sequences that resulted in different image resolution were designed on the Pulse Sequence Designer. The GRE pulse sequences were designed with TR 10ms, whereas the RF pulse was a three lobe sinc-shaped pulse of 3 ms duration and 15° flip angle. A software crusher pulse was applied at the end of each TR. The time-step (temporal resolution) of the pulse sequence was 1 μs, the bandwidth of the receiver was 100 kHz, the field-of-view (FOV) was 40 cm x 40 cm, and the slice thickness was 10 mm. The static magnetic field was set to 1.5 T and the maximum gradient strength was set to 40 mT/m. The ramps of all gradients were considered as steep steps.

For the first GRE pulse sequence, the k-space-matrix was 128 x 128, the TE was 2.23 ms, and the total duration of the pulse sequence 1.28 s; for the second GRE pulse sequence, the k-space-matrix was 256 x 256, the TE was 2.94 ms, and the total duration of the pulse sequence 2.56 s; and for the third GRE pulse sequence, the k-space-matrix was 512 x 512, the TE was 4.43 ms, and the total duration of the pulse sequence 5.12 s. The size of the k-space matrix, the FOV, the slice thickness, the receiver-bandwidth, the gyromagnetic ratio (gamma) and the maximum gradient strength were defined as global parameters in order to be readable by all the objects within the pulse sequence.

Functional programming was applied during the design of the first group of blocks and the addition of the RF, gradient and acquisition objects. For this purpose, some attributes of the objects were defined as functions of the global parameters, other objects’ attributes and the unique increasing ID of the group of blocks. A detailed description of the design of the GRE pulse sequence on the pulse sequence designer is given in S1 Appendix.

#### 2.3.2 MR Imaging mode: coreMRI as a virtual MRI scanner

In order to investigate the utilization of the coreMRI simulation platform as a virtual MRI scanner that simulates the entire MRI process, the three GRE pulse sequences previously generated by the Pulse Sequence Designer (previous section) were applied on an anatomical model of the human brain and the images were reconstructed on Gadgetron.

The anatomical model of the human brain used for simulations was the discrete version of a brain phantom available online by the McConnell Brain Imaging Center at McGill University (Montreal, QC, Canada) [23, 24]. The discrete version of the anatomical model supported only one tissue per position in the 3D space. The 3-D digital brain phantom consisted of 7 different tissue types (connective tissue, fat, glial matter, grey matter, muscle and skin, skull, and white matter) and every tissue type was assigned with its own relaxation times T1 and T2. The size of this model was 18.1 cm x 21.7 cm x 18.1 cm (X x Y x Z) and consisted of 2 380 471 isochromats. The isocenter was placed in the center of the anatomical model and the simulation performed on the entire volume of the anatomical model (i.e. not only within the slice of interest). The simulated MR images were generated with no noise, with Gaussian distributed noise on each signal of the quadrature detector with zero mean and standard deviation equal to 20, and with Gaussian distributed noise on each signal of the quadrature detector with zero mean and standard deviation equal to 40.

To investigate the effect that the volume of the anatomical model has on the reconstructed images, the 512 x 512 GRE pulse sequences was also applied 1) only on the spins located at the z-position of the isocenter (21 113 isochromats) and 2) only on the spins located up to 3 mm (slice thickness 6mm) away from the z-position of the isocenter (147 235 isochromats). No noise was added in these images.

All images were reconstructed on the Gadgetron reconstruction framework using a simple 2D Fourier-Transform (FT) MRI reconstruction as it was described by the Gadgetron configuration file default.xml. The simulations were performed on a p2.16xlarge instance type provided by Amazon AWS equipped with 16 GPUs (NVIDIA K80).

#### 2.3.3 MR Imaging mode: coreMRI for signal simulation purposes

To demonstrate the utilization of coreMRI for signal simulation purposes, a MOdified Look-Locker Inversion recovery (MOLLI) pulse sequence [25] and a computer model of two cylindrical phantoms were developed offline and uploaded to the cloud through the frontend of the coreMRI platform.

The pulse sequence parameters were the following: the TR/TE was 2.06/1.03 ms, the bSSFP readout used a 490 μs sinc-shaped RF pulse with 6 mm slice thickness and 35° excitation flip angle, the IR pulse was a hyperbolic secant adiabatic pulse with 4.74 ms duration, a linear ramp-up preparation of 10 pulses was used to reach steady state prior to the bSSFP readout, the field of view was 360 (FE) x 270 (PE) mm, the scan matrix size was 165 (FE) x 123 (PE), the receiver BW was 200 kHz, and the acquisition scheme was 5(3p)3. The total number of time-steps was 3,300,000.

The computer model consisted of two cylindrical phantoms of radius equal to 50mm and height equal to 2mm each. The left cylinder was assigned with T1 = 900 ms and T2 = 50 ms representing the relaxation times of normal myocardium [26, 27], whereas the right cylinder was assigned with T1 = 1500 ms and T2 = 250 ms representing the relaxation times of blood pool [28, 29]. The isocenter was placed at (0,0,0), and Gaussian distributed noise was introduced on each signal of the quadrature detector with zero mean and standard deviation equal to 20.

The raw data of the simulation were downloaded through the frontend of the coreMRI platform and reconstructed offline using a simple Fourier transform. A MOLLI T1 map was generated and the quantified T1 values were compared against the true values. The simulations were performed on a p2.xlarge instance type provided by Amazon AWS equipped with 1 GPU (NVIDIA K80).

#### 2.3.4 MR Imaging mode: Magnetization transfer

A generalized exchange model that simulated Magnetization Transfer (MT) was developed in coreMRI. This model was based on the solutions of the Bloch-McConnell equations [30] at every point of the anatomical object and at each time point of the pulse sequence. To demonstrate the effect of MT on T1 mapping, the MOLLI pulse sequence (Section 2.3.3) was applied on a computer model both with and without the MT model.

The computer model consisted of three cylinders of radius equal to 50 mm each. The height of both the cylindrical phantoms was selected equal to 2 mm to reduce the total number of isochromats in the computer model and, in turn, to improve the performance of the simulation. The three cylinders represented agar phantoms with an increasing macromolecular content (2%, 4% and 8% agar concentration). The physical properties of these computer phantoms are shown in Table 1 [20, 31]. The raw data of the simulations were downloaded through the frontend of the coreMRI platform and reconstructed offline using a simple Fourier transform in MATLAB. MOLLI T1 maps were generated for both cases (with and without MT model) and the quantified T1 values were compared against the true values. The simulations were performed on a p2.xlarge instance equipped with 1 GPU (NVIDIA K80).

**Table 1:** 

*Phantom*	*T1 (ms)*	*T2 (ms)*
*Agar 2%*	2294	71
*Agar4%*	1735	37
*Agar 8%*	1218	19

Physical properties of three computer models that represent phantoms with an increased macromolecular content (2%, 4% and 8% agar concentration). T1 and T2 values were taken from the literature [31].

#### 2.3.5 MR imaging mode: Motion

**Fig 4:**
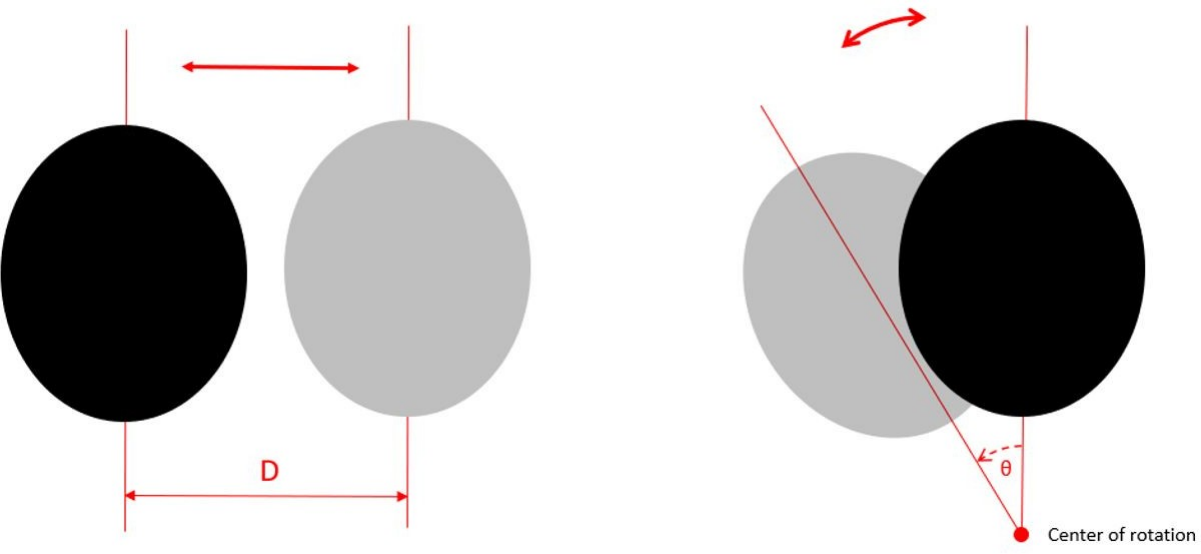
Graphical representation of the two motion models utilized in this study. Translational motion model on the left, rotational motion model on the right.

To simulate more realistic aspects of the MRI experiment and to investigate the performance of the coreMRI platform on a heavy computational environment, two rigid-body motion models were utilized in this study, a translational motion model and a rotational motion model. The translational motion model allowed the translation of the anatomical model along a single axis by a distance D from the initial position with a frequency given in Hz. The rotational motion model allowed the rotation in the XY-plane of the anatomical model around a center of rotation by a rotational angle θ from the initial position with a frequency given in Hz. The rotational motion model was given by:

$$\left(\begin{array}{c} x^{\prime }\\ y^{\prime } \end{array}\right)=\left(\begin{array}{cc} \cos\theta & -\sin\theta \\ \sin\theta & \cos\theta \end{array}\right)\left(\begin{array}{c} x-x_{center}\\ y-y_{center} \end{array}\right)+\left(\begin{array}{c} x_{center}\\ y_{center} \end{array}\right)$$

where x’ and y’ were the new coordinates of each point after rotation around a center of rotation with coordinates x_center_ and y_center_. A graphical representation of the two motion models is given in Fig 4. The displacement of the anatomical model isochromats from their initial position was calculated within the computational kernel of the coreMRI simulation platform for every time-step of the pulse sequence.

For both motion models, the 512 x 512 GRE pulse sequence (section 2.3.1) was applied on the entire volume of the McGill anatomical model of the human brain. For translational-motion simulations, translational motion was induced on the three axes separately for a 5 cm distance and frequency 1Hz. For rotational-motion simulations, rotational motion was induced for 1) a rotational angle of 20^o^ and frequency 1Hz, 2) a rotational angle of 10^o^ and frequency 1Hz, and 3) a rotational angle of 10^o^ and frequency 3Hz. The simulated MR images were generated with no noise. The simulations were performed on a p2.xlarge instance equipped with 1 GPU (NVIDIA K80).

For comparison purposes, a similar GRE pulse sequence was applied on a healthy volunteer with no medical history (female, age 29 years). The healthy volunteer was instructed to rotate her head within the head-coil with a rotational angle of approximately 10^o^ and frequency approximately 2 Hz. The study was approved by the local ethics committee (The Regional Ethics Committee, Lund, Sweden) and the subject provided written consent.

#### 2.3.6 MR Imaging mode: B0 inhomogeneity

**Fig 5:**
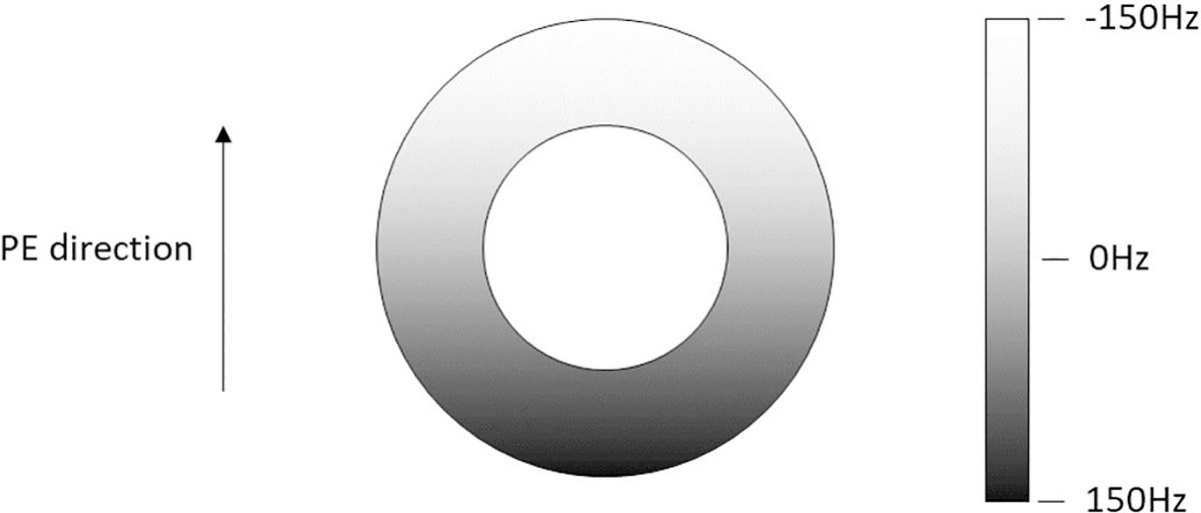
Computer model with a linear B0 inhomogeneity map. Computer model of a cylinder with a linear B0 inhomogeneity map of ±150 Hz across its diameter along the PE direction.

**Fig 6:**
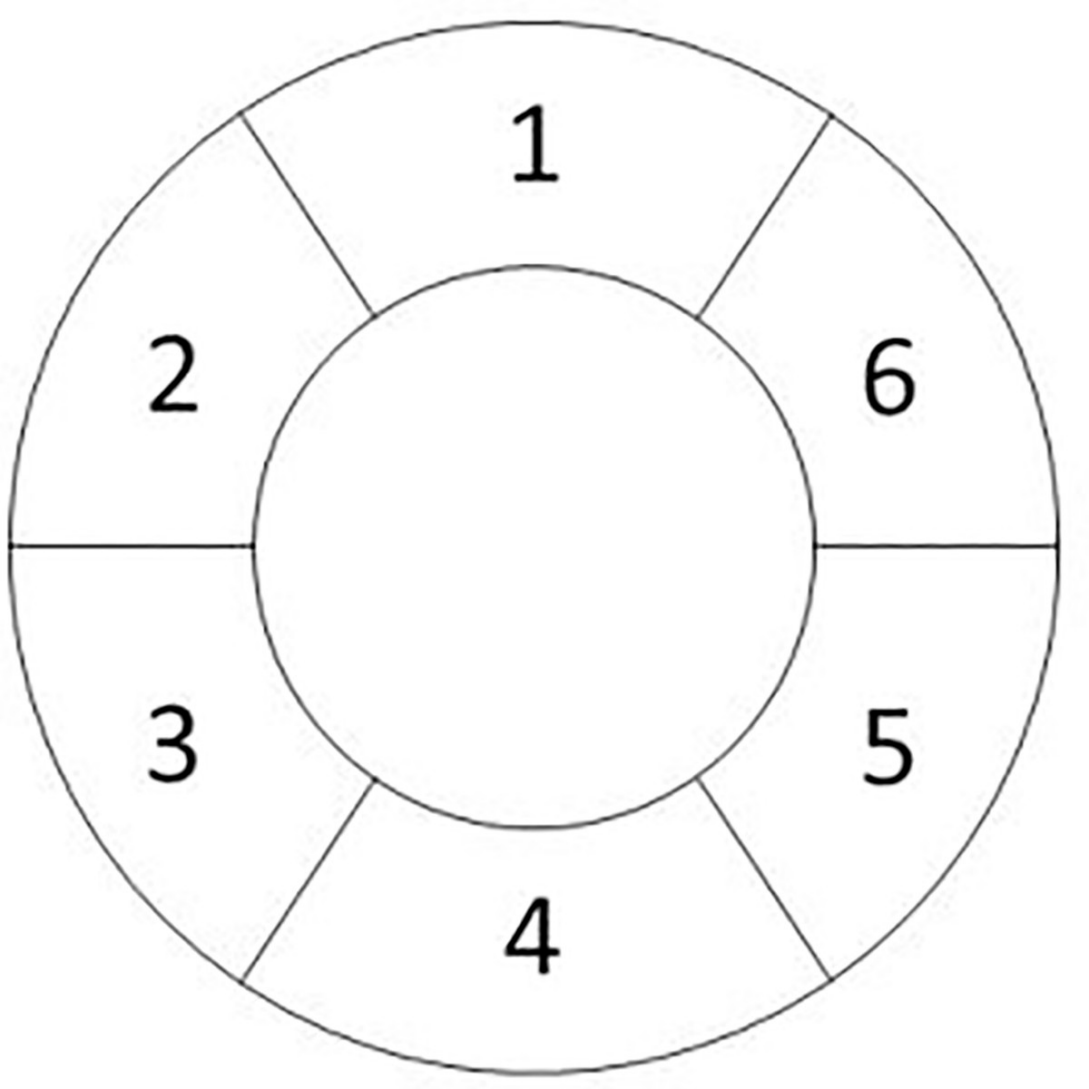
Computer phantom segmentation. Segmentation of the computer phantom for measuring the T1 values from the simulated MR images.

The structure of the anatomical model allowed the introduction of a main-magnetic-field (B0) inhomogeneity map which held the strength of the effective magnetic field that applied on each individual isochromat of the anatomical model. To demonstrate the incorporation of the B0 inhomogeneity map into the coreMRI simulation, the effect of a linear B0 inhomogeneity on T1 mapping was investigated. For this purpose, the MOLLI pulse sequence (Section 2.3.3) was applied on a computer model of a single hollow cylinder with relaxation times (T1 = 900 ms and T2 = 50 ms) similar to the ones reported in the literature for normal myocardium at 1.5 T [26, 27]. The hollow cylinder had a diameter equal to 56 mm and a wall thickness equal to 8 mm representing approximate dimensions of a normal left ventricle [32, 33]. A linear inhomogeneity map that induced a B0 variation of ±150 Hz across the phantom diameter was introduced along the PE direction (Fig 5), covering the maximum B0 variation over the left ventricle of the heart as reported in literature [34]. The T1 values from ROIs within the center of 6 segments (Fig 6) around the phantom were quantified and compared against the true T1 value. The simulations were performed on a p2.xlarge instance equipped with 1 GPU (NVIDIA K80).

In a similar manner, a second experiment was performed to demonstrate how close MR simulations are to real conditions. For this purpose, a phantom (plastic bottle of 500 ml) with target T1 and T2 values close to the relaxations constants of native myocardium was prepared in the lab. Agarose powder was weighed and dissolved in distilled water and the proper amount of 10 mM CuSO4 was added. The mixture was heated, poured into a separate container and left to reach the MR scanner room temperature. The phantom was placed in the scanner in an upright position (long axis of phantom along the posterior-anterior direction) and the T1 and T2 reference values of the phantom were measured. B0 variation of ±150Hz across the phantom diameter (along the z direction) was induced by adding a voltage offset to the first order Z-shim gradient (along the read direction) during imaging and a MOLLI pulse sequence was acquired (TR/TE selected to be the lowest available 2.46/1.23 ms, the bSSFP readout used a 490 μs sinc-shaped RF pulse with 6 mm slice thickness and 35° excitation flip angle, the IR pulse was a tan/tanh adiabatic pulse with 2.56 ms duration, a linear ramp-up preparation of 10 pulses was used to reach steady state prior to the bSSFP readout, the field of view was 360 (FE) x 270 (PE) mm, the scan matrix size was 160 (FE) x 120 (PE), the receiver BW was 200 kHz, and the acquisition scheme was 5(3p)3). B0-maps were estimated on a pixel-wise basis using a multi-echo gradient-recalled echo sequence using six echo times ranging from 4.1–28.5ms, ΔTE 4.9ms, TR 500ms, flip-angle 50° and monopolar readout gradients.

A computer model of the phantom (identical diameter, reference T1 and T2 values, voxel size 0.05mm^3^) was developed and a linear inhomogeneity map that induced a B0 variation of ±150 Hz across the phantom diameter was introduced along the PE direction. The MOLLI pulse sequence (Section 2.3.3) was applied on the computer model of the phantom and the variation of the mean T1 values from three ROIs that were placed along the direction of the induced B0 inhomogeneity were compared against the real MR experiment. A second set of experiments (MR scanner and simulation) was performed without the application of B0 inhomogeneity.

#### 2.3.7 MR Imaging mode: Multiple-element receiver coil

**Fig 7:**
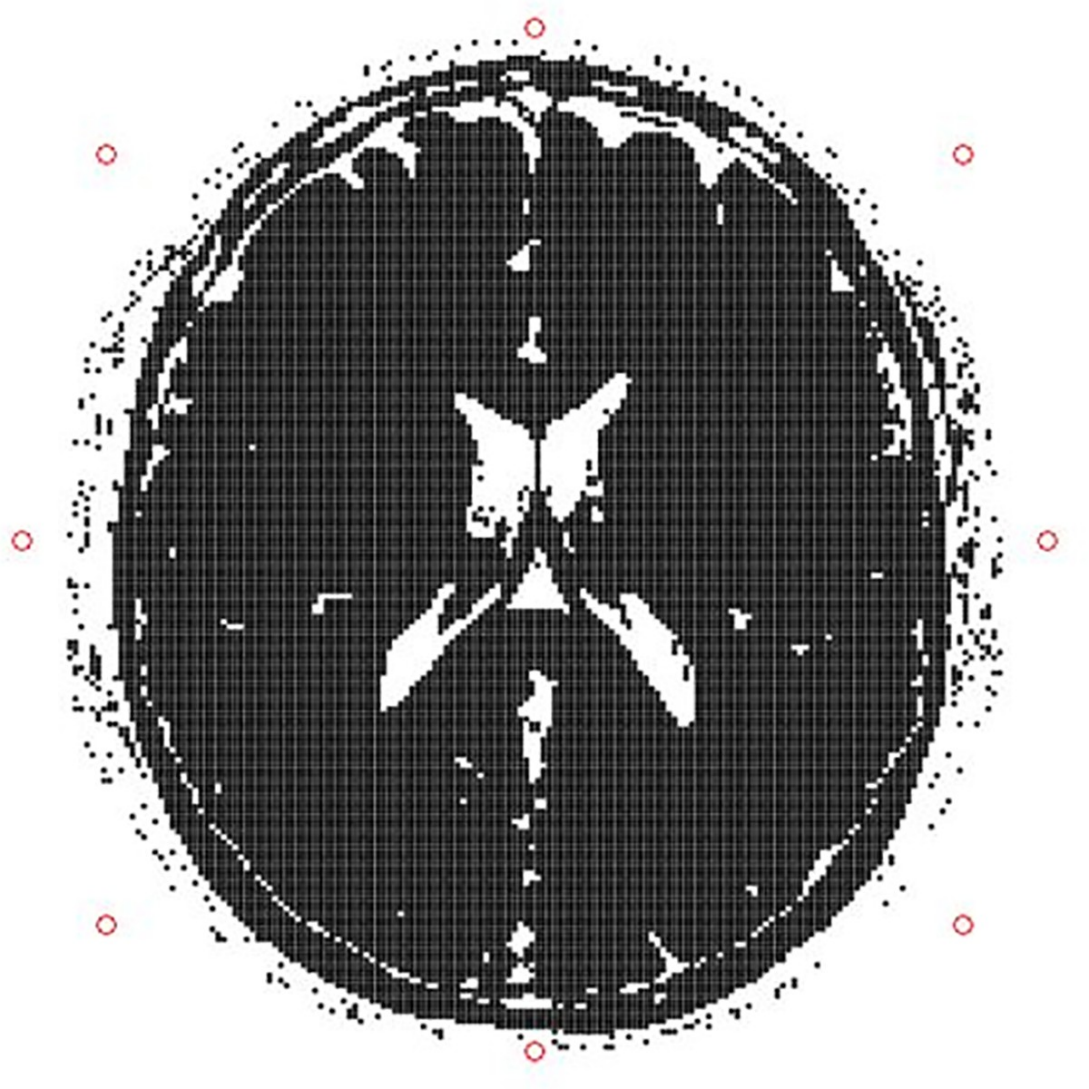
Position of the coil elements. Position of the eight circular coil elements around the transversal slice of the McGill anatomical model of the human brain at z position of 9.05 cm.

The structure of the anatomical model allowed the simulation of a multiple-element receiver coil through the introduction of the sensitivity maps of every coil element. Eight circular coil elements of 60 mm radius each were placed in the three-dimensional space around the McGill anatomical model of the human brain, around the transversal slice at z position equal to 9.05 cm, as shown in Fig 7. The three-dimensional sensitivity maps of the coil elements were calculated using the Biot-Savart law with a spatial resolution identical to the resolution of the anatomical model. The anatomical model of the human brain (including the coil sensitivity maps) was uploaded on the coreMRI platform and the 512 x 512 GRE pulse sequence was applied on the entire volume of the anatomical model. The simulation was performed on a p2.16xlarge instance equipped with 16 GPUs (NVIDIA K80).

#### 2.3.8 Quantitative MR mode: Simulation-based quantitative MR methods (SQUAREMR)

To investigate the utilization of coreMRI in simulation-based quantitative MR techniques (such as SQUAREMR [15]), the coreMRI simulation platform was used for the generation of extensive databases of simulated MR signals. A MOLLI pulse sequence was first applied on a healthy human volunteer on a MAGNETOM Aera 1.5 T scanner (Siemens Healthcare, Erlangen, Germany) and then extracted from the MR scanner and simulated on coreMRI for a wide range of T1 and T2 values [15, 31]. One SASHA experiment [34] using a prototype sequence and a T2prep-bSSFP experiment [35] as reference for T1 and T2 mapping, respectively, were acquired. The study was approved by the local ethics committee (The Regional Ethics Committee, Lund, Sweden) and the subject provided written consent.

The MOLLI pulse sequence parameters were the following: an acquisition scheme 5s(3s)3s was used, the IR pulse was a 5.12 ms tangent/hyperbolic tangent adiabatic, the inversion time (TI) range was 103–183 ms, the bSSFP readout used a 1050 μs sinc-shaped RF pulse with 6 mm slice thickness and 70^o^ excitation flip angle, the field-of-view (FOV) was 360 mm × 270 mm, the acquisition matrix was 192 ×144, the receiver bandwidth (rBW) was 1085 Hz/pixel, the TE/TR were 1.29/2.95 ms, whereas phase partial Fourier 7/8, GRAPPA acceleration factor of 2 with 36 reference lines acquired at the end of the MOLLI pulse sequence (GRE-separate), linear k-space trajectory and linear ramp up preparation of 5 RF pulses were utilized. The bSSFP acquisition window was 185 ms and the total duration of the MOLLI acquisition was 11 s. The MOLLI pulse sequence was extracted through the sequence development environment from the IDEA framework (Siemens Healthcare, Erlangen, Germany) as a .dsv file format and converted to a coreMRI compatible format. The effective inversion times of the single-shot images [15] were defined and the pulse sequence was uploaded to the cloud through the frontend of the coreMRI platform.

The pulse sequence was simulated on a spin population (138,621 spins) covering a large range of physiological combinations of native myocardial T1 and T2 values. T1 and T2 values of 700–1500 ms and 20– 100 ms, respectively, were simulated with a T1 step of 5 ms and a T2 step of 2 ms. The excitation slice profile was simulated on 21 spins across the slice-selection direction [15]. The pulse sequence consisted of 1,286,041 discrete time-steps and a database of 6,601 entries, each with 8 points, was generated. The simulation was performed on a p2.xlarge instance equipped with 1 GPU (NVIDIA K80).

T1 and T2 maps from the healthy volunteer with no medical history (female, age 22 years) were produced using the SQUAREMR method [15] and the SQUAREMR-based quantified T1 and T2 values from a septal ROI were compared against the corresponding values resulted from conventional T1 and T2 mapping techniques (MOLLI [25] and SASHA [35] for T1 mapping and T2prep-bSSFP [36] for T2 mapping). An extensive validation of the SQUAREMR technique on phantoms and healthy volunteers is available in the literature [31].

#### 2.3.9 coreMRI performance assessment

To demonstrate the performance and scalability of coreMRI on multi-GPU configurations on the cloud, the execution times of the computational kernel were recorded for eight (8) different types of GPU-equipped instances (g2, g3 and p2 family types of GPU-equipped instances), two (2) different types of experiments (MR Imaging and quantitative MR) and several different sizes of simulations (size of anatomical model and size of pulse sequence).

For the purpose of MR imaging, the 512 x 512 GRE pulse sequence (section 2.3.1) was applied on the McGill human brain phantom (section 2.3.2). The GRE pulse sequence was applied in its uncompressed and compressed versions (fast algorithm [13]) on both the entire volume and a single slice of the McGill human brain phantom. The fast algorithm allowed for a variable time step size during times when no changes occurred within the pulse sequence and when the acquisition window was off. These combinations resulted in four (4) different sets of simulations in total: 1) Uncompressed GRE pulse sequence on the entire volume of the anatomical model, 2) Compressed GRE pulse sequence on the entire anatomical model, 3) Uncompressed GRE pulse sequence on a single slice of the anatomical model and 4) Compressed GRE pulse sequence on a single slice of the anatomical model. The number of the total time-steps of the pulse sequence was 5,120,000 for its uncompressed version (fast algorithm disabled) and 3,651,072 for its compressed version (fast algorithm enabled). The entire anatomical model consisted of 2,380 471 isochromats, whereas the single slice consisted of 21,113 isochromats. Last, for comparison purposes, the simulation of the compressed version of the 512 x 512 GRE pulse sequence on the entire volume of the McGill human brain phantom was also performed using another simulation platform, MRISIMUL [12, 13], on an in-house, single GPU computer system equipped with a TESLA C2070 GPU card (448 CUDA cores and 6GB GPU memory).

For the purpose of quantitative MR mode, the compressed version (fast algorithm [13]) of MOLLI pulse sequence was utilized on a population of spins and one database of simulated MR signals was generated for the range of physiological combinations of native myocardial T1 and T2 values described in section 2.3.8 (total database entries 6,601).

For every family type of the GPU-equipped instances (g2, g3 and p2), the speedup was calculated against the single-GPU configuration of the g2 family (g2.2xlarge) which is considered as the earliest/oldest available GPU-based instance provided by Amazon AWS. The execution times of the computational kernel were measured in seconds.

## 3 Results

### 3.1 MR imaging mode

#### 3.1.1 Pulse sequence designer

**Fig 8:**
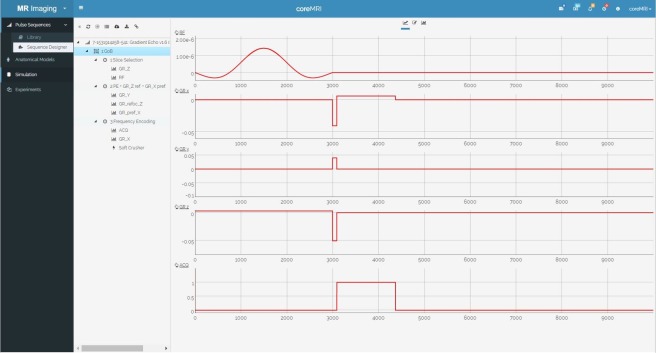
Pulse sequence designer. The web-application of the Pulse Sequence Designer and the graphical representation of the first Group of Blocks (GoB) of the 128x128 Gradient Echo pulse sequence.

**Fig 9:**
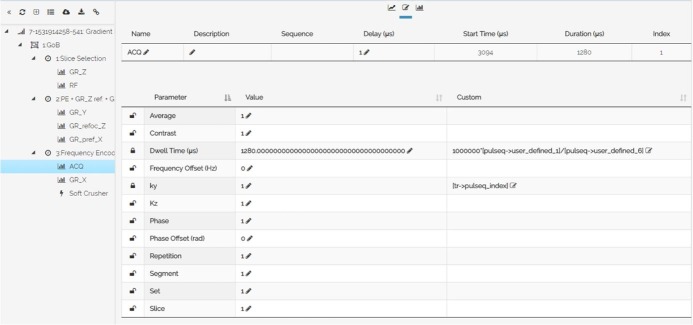
Pulse sequence designer. The web-application of the Pulse Sequence Designer and the presentation of the acquisition object’s parameters organized in a table form. The fourth column (Custom) of the table shows the functional programming that was applied during the design of this object.

**Fig 10:**
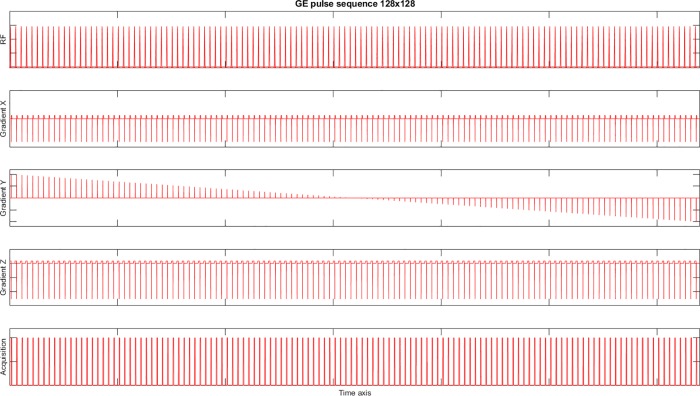
Illustration of the entire 128x128 Gradient Echo pulse sequence. This figure was generated offline. The first graph (top to bottom) presents the magnitude of the RF pulses, the second to fourth graphs present the magnitude of the gradients applied on X, Y and Z axis, respectively, whereas the fifth graph presents the acquisition objects. (A MATLAB script to display the entire pulse sequence is available at this link: www.coremri.org/index.php/documentation/29-pulse-sequence-gui-plot-the-entire-pulse-sequence).

The graphical representation of the first Group of Blocks (GoB) of the 128x128 Gradient Echo pulse sequence is shown in Fig 8. This GoB represents the first repetition time (TR). In the tree-view interface, on the left side of the pulse sequence designer, the GoB is organized in three blocks, and these blocks hold RF, gradient, software crushers and acquisition objects. On the right side of the pulse sequence designer, the graphical representation of the selected GoB is shown. The graphical representation is divided into the following five diagrams (from top to bottom): RF objects, gradient objects on X axis (frequency-encoding direction), gradient objects on Y axis (phase-encoding direction), gradient objects on Z axis (slice-selection direction) and acquisition objects. Fig 9 shows the acquisition object’s parameters organized in a table form, whereas the fourth column (Custom) of the table shows the functional programming that was applied during the design of this object. Last, Fig 10 shows the design of the entire GRE pulse sequence.

#### 3.1.2 Virtual MR scanner

**Fig 11:**
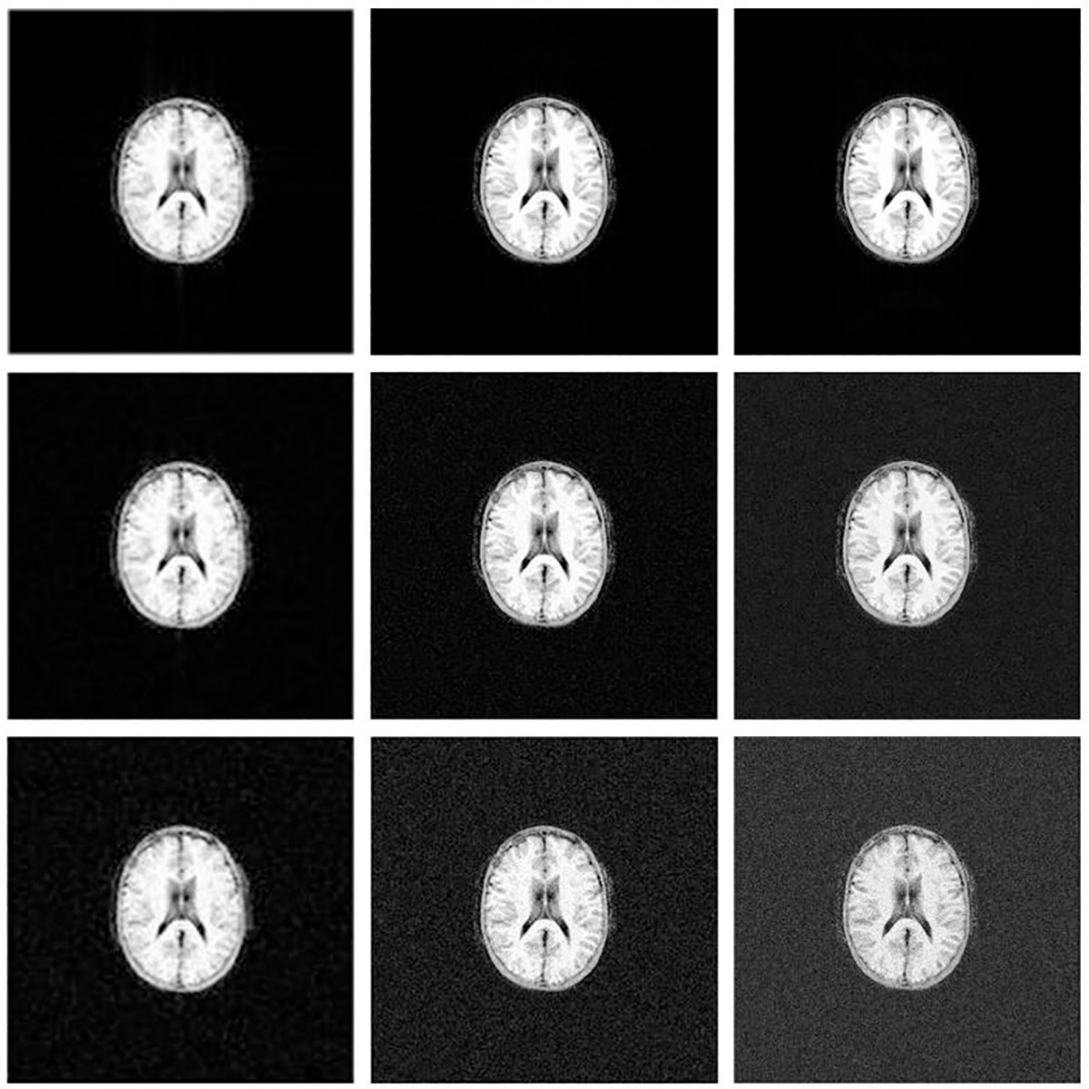
Simulated MR images after the application of GRE pulse sequences on the entire volume of the McGill anatomical model of the human brain and reconstructed on Gadgetron. Left column presents the simulated MR images from a 128x128 GRE pulse sequence, center column presents the simulated MR images from a 256x256 GRE pulse sequence, and right column presents the simulated MR images from a 512x512 GRE pulse sequence. The first row shows the GRE images with no noise, the second row shows the same GRE images with Gaussian distributed noise with zero mean and standard deviation equal to 20, whereas the third row shows the same GRE images with Gaussian distributed noise with zero mean and standard deviation equal to 40. The contrast of each image was adjusted to fit the image data range, with a 2% padding at the upper and lower bounds. The kernel execution time on a p2.16xlarge instance was 99s for the 128x128 GRE pulse sequence, 209s for the 256x256 GRE pulse sequence and 457s for the 512x512 GRE pulse sequence.

Fig 11 shows GRE images obtained with the simulator after the application of the GRE pulse sequences (128x128, 256x256 and 512x512) on the entire volume of the McGill anatomical model of the human brain and reconstructed on Gadgetron. The first row of Fig 11 shows the GRE images with no noise, the second row shows the same GRE images with Gaussian distributed noise on each signal of the quadrature detector with zero mean and standard deviation equal to 20, whereas the third row shows the same GRE images with Gaussian distributed noise on each signal of the quadrature detector with zero mean and standard deviation equal to 40.

**Fig 12:**
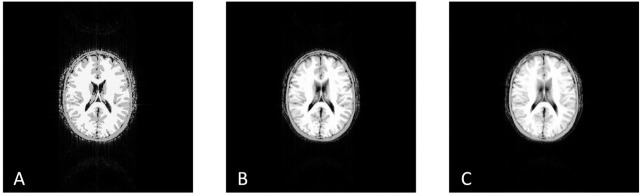
Noiseless GRE images obtained with the simulator after the application of the 512x512 GRE pulse sequence. A) only on the spins located at the z-position of the isocenter B) only on the spins located ±3 mm (slice thickness 6mm) away from the z-position of the isocenter, and C) on the entire volume of the anatomical model. The kernel execution time on a p2.xlarge instance was 292 s for case A, 451 s for case B and 4,801 s for case C. Note the different appearance of the simulated MR images for the different volumes of spins of the same anatomical model.

Fig 12 presents the noiseless GRE images obtained with the simulator after the application of the 512x512 GRE pulse sequence A) only on the spins located at the z-position of the isocenter B) only on the spins located up to ±3 mm away from the z-position of the isocenter and C) on the entire volume of the anatomical model. The generated MR images present well the anatomical regions of the computer model of the human brain (compared to Fig 7) and the contrast between the different tissue types as defined by the different tissues characteristics (T1, T2, etc.) and the characteristics of the pulse sequence (TE, TR, etc.).

#### 3.1.3 coreMRI for simulation purposes only

**Fig 13:**
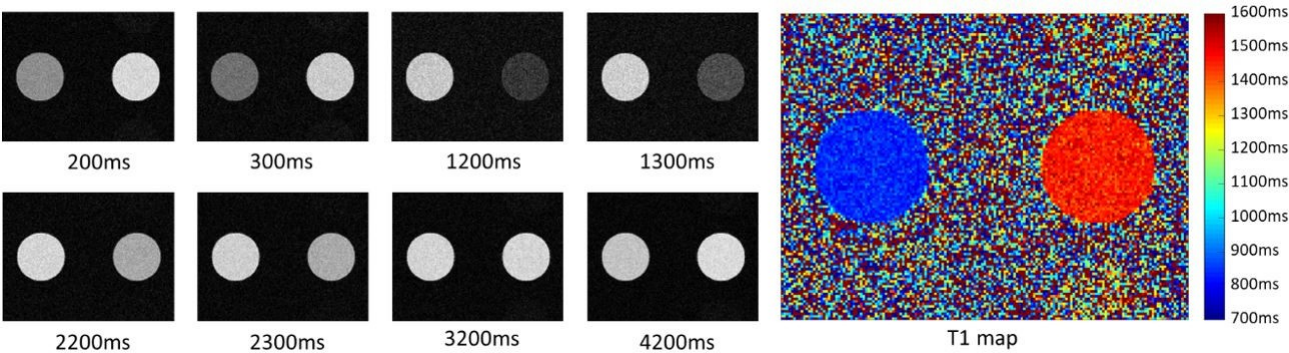
MOLLI pulse sequence images. On the left, magnitude images obtained from MOLLI pulse sequence sorted by inversion time. On the right, the resulting coloured MOLLI T1 map. The kernel execution time on a p2.xlarge instance was 118 s.

Fig 13 depicts the magnitude images sorted by inversion time and the resulting coloured MOLLI T1 map. The average T1 values of the two phantoms were measured equal to 837 ms and 1451 ms. The underestimation of MOLLI on the true T1 values (900 ms and 1500 ms, respectively) agrees with previous studies [15, 28].

#### 3.1.4 Magnetization transfer

**Fig 14:**
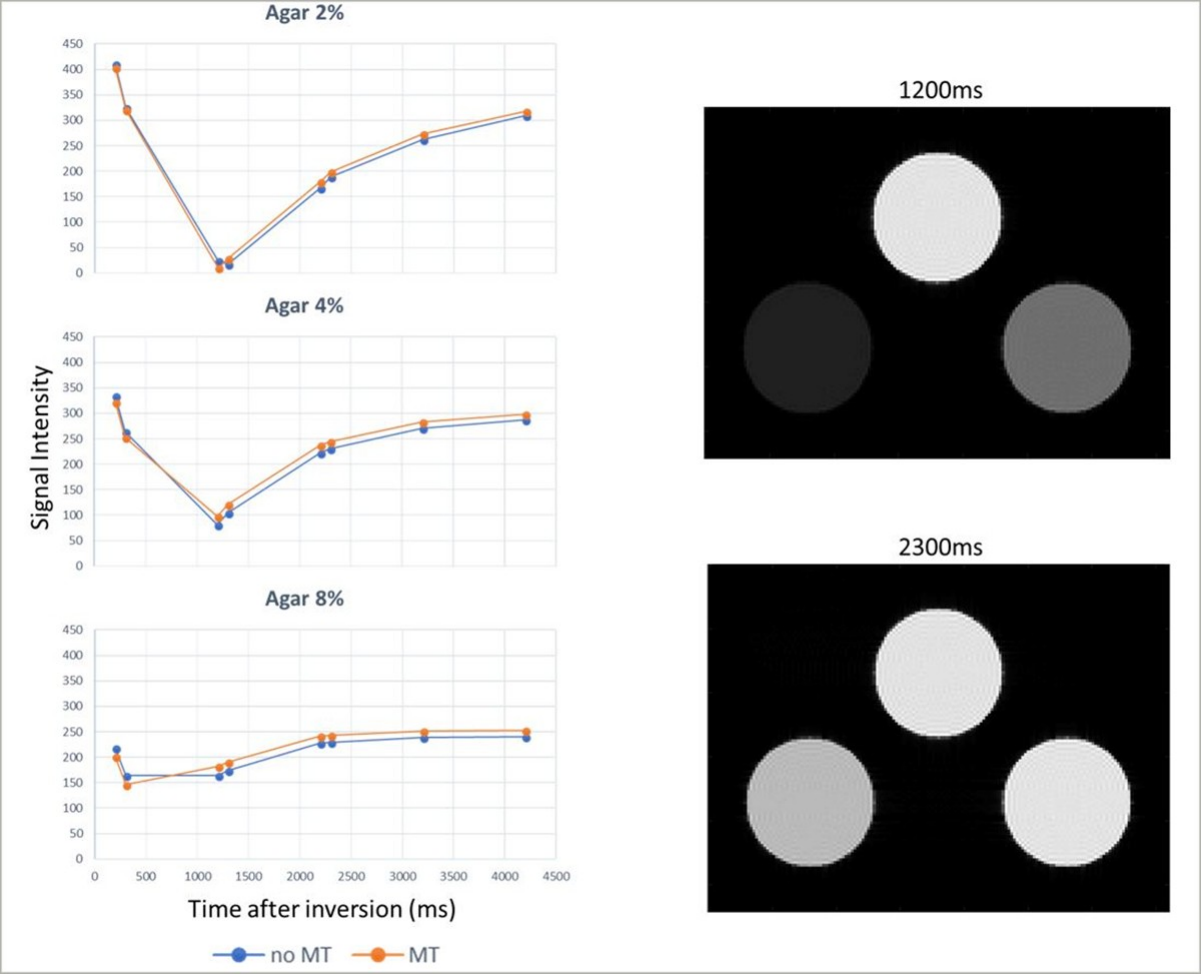
Magnetization transfer. (Left) Signal intensity curves for the three agar computer phantoms: Agar 2% on the left, Agar 4% in the center, and Agar 8% on the right. The curve in blue shows the signal intensity without the simulation of the MT model, whereas the curve in orange shows the corresponding signal intensity with simulation of the MT model. Note the change of the shapes of the inversion recovery curves due to MT. (Right) Magnitude images of the computer model of three cylinders after the application of a MOLLI pulse sequence for inversion times 1200 ms and 2300 ms. The bottom left cylinder represents an Agar phantom with a 2% macromolecular content, the bottom right cylinder represents an Agar phantom with a 4% macromolecular content, whereas the top cylinder represents an Agar phantom with an 8% macromolecular content. The kernel execution time on a p2.xlarge instance was 105 s without the simulation of the MT model and 137 s with the simulation of the MT model.

Fig 14 (right) shows the magnitude images of the three-cylinder phantom setup. The images were obtained from the simulator with the application of the MT model. The signal intensity curves for the three agar phantoms, with and without the simulation of the MT model, are shown in Fig 14 (left) at a simulated heart rate of 60 bpm. The corresponding T1 values were 2031 ms, 1512 ms and 1003 ms for the first case (no MT), and 1920 ms, 1405 ms and 902 ms for the second case (with MT) for the phantoms agar 2%, 4% and 8%, respectively. The bias in T1 estimates due to the MT effect agrees with previous studies [28, 31] that investigate the effect of MT on MOLLI T1 estimates.

#### 3.1.5 Motion

**Fig 15:**
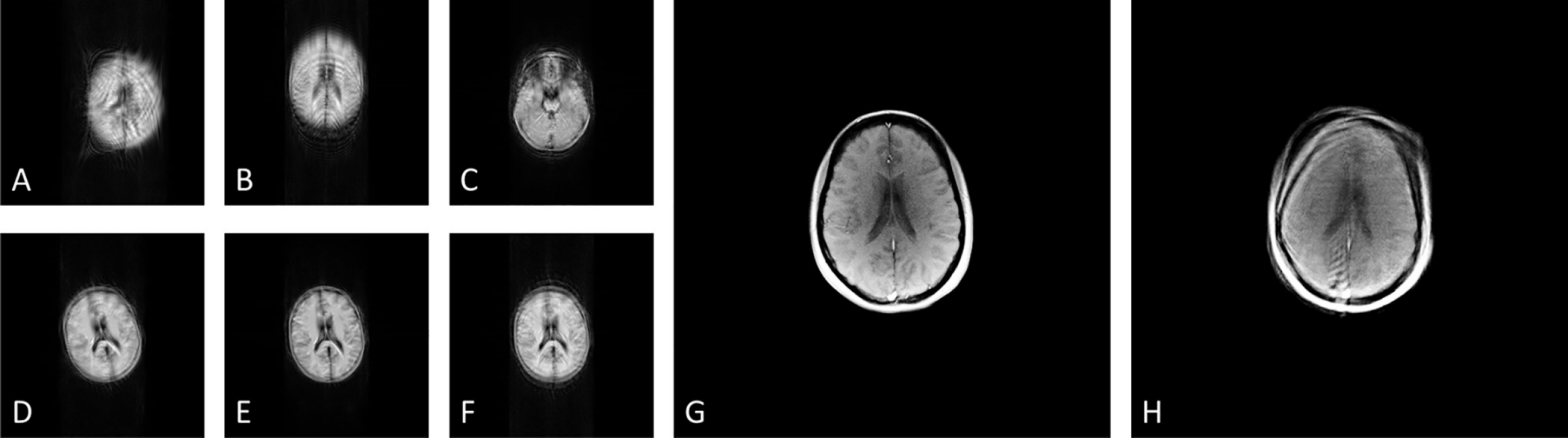
Noiseless GRE images with motion-induced artifacts after the application of the 512x512 GRE pulse sequence on the entire volume of the McGill anatomical model of the human brain. A) Under translational motion of 5cm distance and frequency 1 Hz on x axis, B) under translational motion of 5cm distance and frequency 1 Hz on y axis, C) under translational motion of 5cm distance and frequency 1 Hz on z axis, D) under rotational motion with a rotational angle of 20^o^ and frequency 1 Hz, E) under rotational motion with a rotational angle of 10^o^ and frequency 1 Hz, and F) under rotational motion with a rotational angle of 10^o^ and frequency 3 Hz. Note the motion artifacts in the phase-encoding direction. The kernel execution time on a p2.xlarge instance was 4235 s without the simulation of the motion model, 4408 s for the translational motion model and 4709 s for the rotational motion model. For comparison purposes, Fig H presents the GRE head image that was acquired from a healthy volunteer who was instructed to rotate the head within the head-coil with a rotational angle of approximately 10^o^ and frequency approximately 2 Hz. Motion artifacts are present in the phase-encoding direction (posterior to anterior). Fig G shows the GRE head image that was acquired from the healthy volunteer under no motion.

The motion-induced artifacts on the GRE images of the McGill anatomical model of the human brain are presented in Fig 15. Fig 15A, 15B and 15C illustrate the anatomical model under translational motion of 5 cm distance and frequency 1 Hz on x, y and z axis, respectively, whereas Fig 15D, 15E and 15F illustrate the anatomical model under rotational motion with a rotational angle of 20^o^ and frequency 1 Hz, a rotational angle of 10^o^ and frequency 1 Hz, and a rotational angle of 10^o^ and frequency 3 Hz, respectively. For comparison purposes, Fig 15H presents the GRE head image that was acquired from a healthy volunteer who was instructed to rotate the head within the head-coil with a rotational angle of approximately 10o and frequency approximately 2 Hz. Fig 15G shows the GRE head image that was acquired from the healthy volunteer under no motion.

#### 3.1.6 B0 inhomogeneity

**Fig 16:**
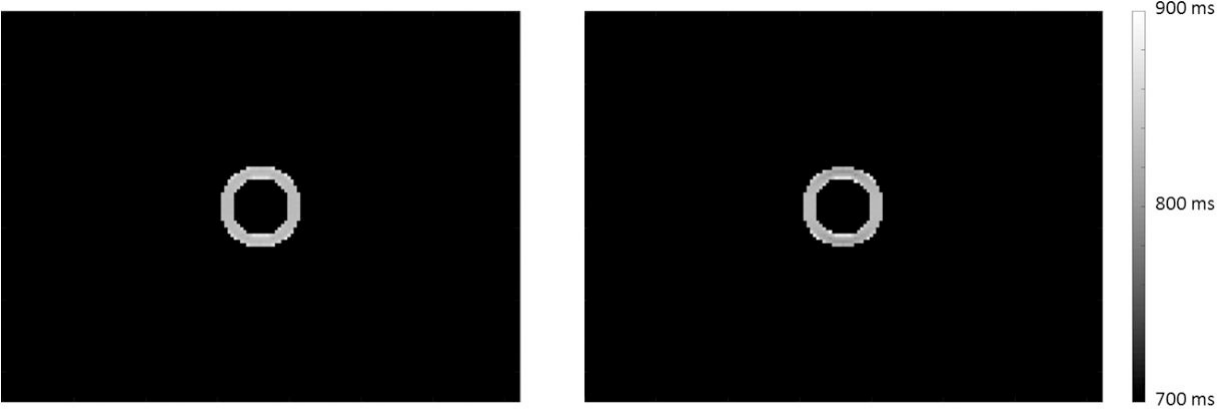
T1 maps of the computer model of the myocardium after the application of the MOLLI pulse sequence. The left image depicts the T1 map without the application of the B0 inhomogeneity map, whereas the right image presents the T1 map with the application of a B0 variation of ±150 Hz across the phantom diameter along the PE direction. Note artifactual local variations in the estimated T1 across the phase-encoding direction on the computer model in the right image. The kernel execution time on a p2.xlarge instance was 115 s with and without the application of the B0 inhomogeneity map.

Fig 16 shows the T1 maps of the computer model of the myocardium. The left image of Fig 16 presents the T1 map obtained from the simulator without the application of the B0 inhomogeneity map, whereas the right image presents the T1 map obtained with the application of a B0 variation of ±150 Hz across the phantom diameter along the PE direction. The measured T1 values from the ROIs within the center of the six segments with and without the B0 variation were 804 ms vs. 831 ms (segment 1), 828 ms vs. 835 ms (segment 2), 826 ms vs. 833 ms (segment 3), 814 ms vs. 835 ms (segment 4), 824 ms vs. 835 ms (segment 5), and 827 ms vs. 834 ms (segment 6). The variation in T1 estimates due to off-resonance is larger in segments 1 and 4 where the B0 variation is stronger. This trend has already been shown in a previous study in the literature [34].

For comparison purposes, a second experiment was performed to demonstrate how close MR simulations are to real conditions. The reference T1 and T2 values of the phantom were 1091 ms and 44 ms respectively. The measured T1 values from the three ROIs that were placed along the direction of the induced B0 inhomogeneity in true and simulated MOLLI images (noiseless) with and without the application of B0 inhomogeneity are presented in Table 2. The T1 measurements in true MOLLI images are close to the T1 measurements in simulated MOLLI images with and without the application of B0 inhomogeneity.

**Table 2:** 

		T1 values (ms, mean±SD)		
		ROI on one side of phantom (negative B0 inhom.)	ROI at the center of phantom	ROI on one side of phantom (positive B0 inhom.)
With B0 inhomogeneity	MR scanner	954±27	1002±8	957±12
	Simulation	956±3	994±9	953±4
Without B0 inhomogeneity	MR scanner	1003±18	1000±6	1014±10
	Simulation	994±1	996±1	994±1

T1 values (in ms, mean±SD) measured from three ROIs that were placed along the direction of the induced B0 inhomogeneity in true and simulated MOLLI images (noiseless) with and without the application of B0 inhomogeneity. The reference T1 and T2 values of the phantom were 1091 ms and 44 ms.

#### 3.1.7 Multiple-element receiver coil

**Fig 17:**
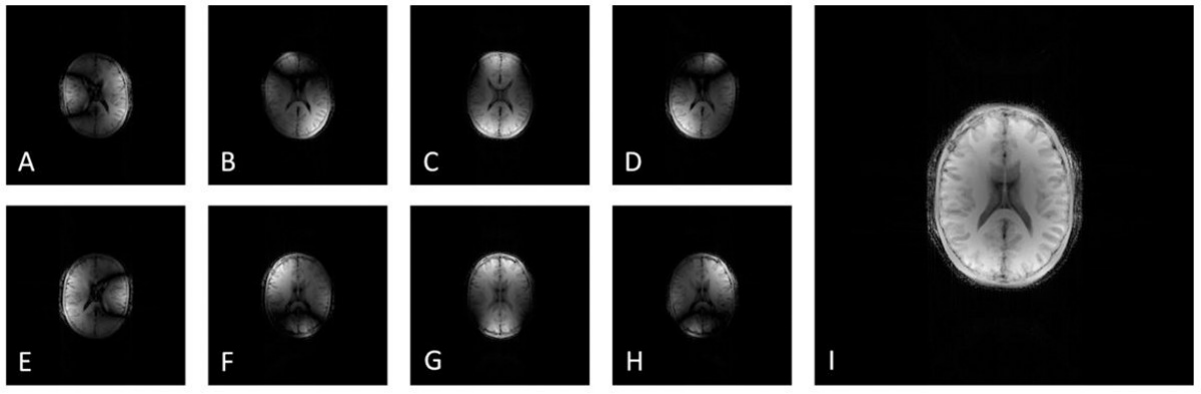
Multiple-element receiver coil. A-H) Noiseless GRE images per coil element after the application of the 512x512 GRE pulse sequence on the entire volume of the McGill human brain phantom. I) The final noiseless reconstructed GRE image after the combination of all the coil-elements. The images were reconstructed on Gadgetron. The kernel execution time on a p2.16xlarge instance was 1177 s.

Fig 17 shows the results obtained with coreMRI and a multiple-element receive configuration with a GRE pulse sequence (512x512) applied on the entire volume of the McGill human brain phantom. Fig 17A–17H illustrate the per-coil-element reconstructed magnitude images, whereas Fig 17I shows the final reconstructed image.

### 3.2 Quantitative MR mode

#### 3.2.1 Simulation-based quantitative MR methods (SQUAREMR)

**Fig 18:**
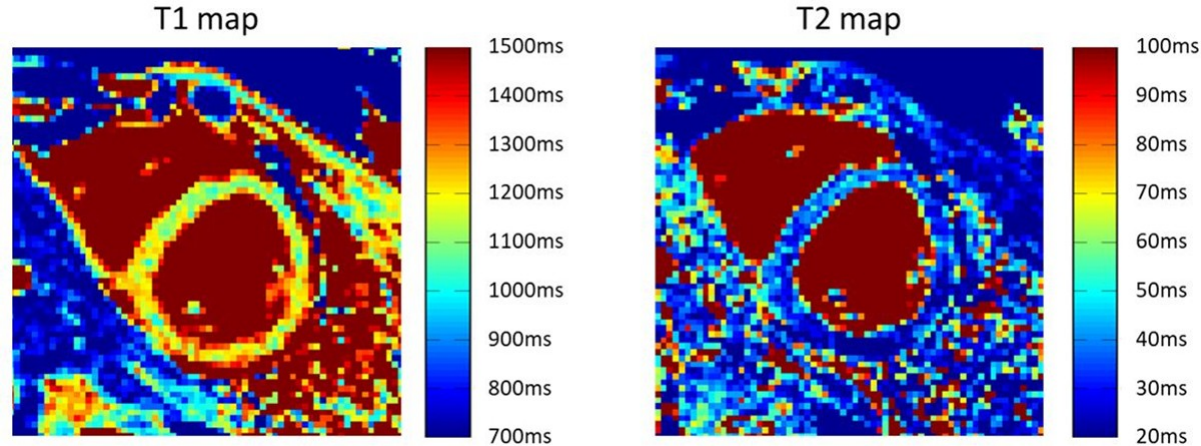
Quantitative measurements using the modified version of the MOLLI pulse sequence and the SQUAREMR method. T1 and T2 maps of a healthy volunteer extracted from the modified version of the MOLLI pulse sequence and the SQUAREMR method [15]. The kernel execution time on a p2.xlarge instance for the generation of database with the simulated MR signals took 154 s.

Fig 18 presents the T1 and T2 maps from the healthy volunteer derived from the SQUAREMR method. The average myocardial T1 from an ROI placed at the septum was 1149±56 ms for SQUAREMR and 1005±23 ms for conventional MOLLI and 1251±70 ms for conventional SASHA, whereas the average myocardial T2 from the same ROI was 43±8 ms for SQUAREMR and 46±2 ms for conventional T2prep-bSSFP.

### 3.3 coreMRI performance assessment

Table 3 displays the kernel execution times for the eight different types of GPU-equipped instances provided by Amazon AWS and for the four different MR Imaging experiment types: 1) Uncompressed GRE pulse sequence on the entire volume of the anatomical model, 2) Compressed GRE pulse sequence on the entire anatomical model, 3) Uncompressed GRE pulse sequence on a single slice of the anatomical model, and 4) Compressed GRE pulse sequence on a single slice of the anatomical model.

**Table 3:** 

			Uncompressed GRE (512x512) on entire volume		Compressed GRE (512x512) on entire volume		Uncompressed GRE (512x512) on single slice		Compressed GRE (512x512) on single slice	
instance-type	GPU-type	no. GPUs	Kernel Time (s)	Speedup	Kernel Time (s)	Speedup	Kernel Time (s)	Speedup	Kernel Time (s)	Speedup
g2.2xlarge	K520	1	54162		50031		2690		2496	
g2.8xlarge	K520	4	13923	3.9	13247	3.8	2550	1.1	2478	1.0
g3.4xlarge	Tesla M60	1	3336	16.2	2605	19.2	194	13.9	150	16.6
g3.8xlarge	Tesla M60	2	1781	30.4	1385	36.1	196	13.7	152	16.4
g3.16xlarge	Tesla M60	4	894	60.6	694	72.1	197	13.7	152	16.4
p2.xlarge	K80	1	4235	12.8	3049	16.4	240	11.2	180	13.9
p2.8xlarge	K80	8	669	81.0	513	97.6	242	11.1	184	13.6
p2.16xlarge	K80	16	454	119.3	345	145.0	245	11.0	184	13.5

Kernel execution times for eight different types of GPU-equipped instances (g2, g3 and p2 family types of GPU-equipped instances) and four different sizes of simulations: 1. Uncompressed GRE pulse sequence on the entire volume of the anatomical model (5,120,000 time-steps and 2,380,471 isochromats), 2. Compressed GRE pulse sequence on the entire anatomical model (3,651,072 time-steps and 2, 380,471 isochromats), 3. Uncompressed GRE pulse sequence on a single slice of the anatomical model (5,120,000 time-steps and 21,113 isochromats), and 4. Compressed GRE pulse sequence on a single slice of the anatomical model (3,651,072 time-steps and 21,113 isochromats). Note that a single-GPU computer configuration performed fast simulations of a large pulse sequence on a single slice of the anatomical model (150 s on g3.4xlarge), whereas a multi-GPU computer performed fast simulations of a large pulse sequence on the entire volume of the anatomical model (345 s on p2.16xlarge).

Table 4 displays the kernel execution times for the eight different types of GPU-equipped instances provided by Amazon AWS and for the application of the compressed MOLLI pulse sequence on the spin population.

**Table 4:** 

			Quantitative MR for T1 and T2 mapping using SQUAREMR	
instance-type	GPU-type	no. GPUs	Kernel Time (s)	Speedup
g2.2xlarge	K520	1	208.42	
g2.8xlarge	K520	4	62.84	3.3
g3.4xlarge	Tesla M60	1	105.12	2.0
g3.8xlarge	Tesla M60	2	56.45	3.7
g3.16xlarge	Tesla M60	4	31.81	6.6
p2.xlarge	K80	1	155.30	1.3
p2.8xlarge	K80	8	28.54	7.3
p2.16xlarge	K80	16	20.12	10.4

Kernel execution times for the eight different types of GPU-equipped instances (g2, g3 and p2 family types of GPU-equipped instances) and for the application of the compressed version of the MOLLI pulse sequence (1,286,041 time-steps) on the population of spins (138,621 isochromats). Note that the generation of the database of simulated MR signals is feasible within clinically acceptable levels (20 s on p2.16xlarge).

The generation of the brain image took 454 s on the 16 GPUs computer system (p2.16xlarge type equipped with 16 K80 GPU devices) using the uncompressed version of the pulse sequence on the entire volume of the anatomical model (heavy computational environment) and 150 s on the single GPU computer system (g3.4xlarge type equipped with 1 Tesla M60 GPU device) using the compressed version of the pulse sequence on a single slice of the anatomical model (simple computational environment). The generation of the database of simulated MR signals took 20 s using the compressed version of the MOLLI pulse sequence on the 16-GPU computer system (p2.16xlarge type equipped with 16 K80 GPU devices). These results point out that a single-GPU computer configuration performs well on a single slice of the anatomical model, whereas a multi-GPU computer configuration performs similarly well on heavier computational environments and within clinically acceptable levels in quantitative MRI methods. Last, for comparison purposes, the simulation of the compressed version of the 512 x 512 GRE pulse sequence on the entire volume of the McGill human brain phantom with the use of the simulation platform MRISIMUL [12, 13] took 1693 s on an in-house, single GPU, computer system (TESLA C2070 GPU card).

## 4 Discussion

An advanced and high-performance MR simulation platform that is delivered as a web service through the cloud by utilizing a cloud-and-GPU-based infrastructure, was presented in this study. The coreMRI simulation platform is publicly available to the entire MR community (www.coremri.org) and allows its users to configure advanced MRI experiments without the constraints imposed by experimentation in a true MRI scanner (such as time constraint and limited availability of MR scanners), without upfront investment for purchasing advanced computer systems and without any user expertise on computer programming or MR physics.

In this study, the coreMRI simulation platform was designed and developed as a web service that provided a web-based interface to the cloud-and-GPU-based infrastructure where the computationally demanding MR simulations were performed. Two simulation modes were considered that allowed the coreMRI simulation platform to be utilized either as a virtual MR scanner that simulates the entire MRI process (from pulse sequence development and selection of the anatomical model to signal generation and image formation) or as a generator of databases of simulated MR signals to be used in simulation-based quantitative MR methods, such as SQUAREMR [14, 15]. Moreover, the coreMRI simulation platform was based on a flexible, modular design that allowed individual modules to be added in the main simulation framework. In this study, the Gadgetron reconstruction framework was added as a reconstruction module in the coreMRI workflow, whereas a new module (Pulse Sequence Designer) that provided a web-application for pulse sequence design was developed and added in the coreMRI simulation platform.

The different types and sources of pulse sequences and anatomical models that were utilized in this study showed the flexibility that the current design of the coreMRI simulation platform offered to the users. Three types and sources of pulse sequences were used in the current work: 1) Gradient Echo pulse sequences that were developed in the Pulse Sequence Designer of the coreMRI simulation platform, 2) a MOLLI pulse sequence that was developed offline and uploaded to the coreMRI platform, and 3) a MOLLI pulse sequence that was extracted from a true MR scanner and uploaded to the coreMRI platform. The pulse sequences were applied on a 3-D digital phantom of the human brain and on custom-made anatomical models, whereas other true aspects of MR Imaging, such as magnetization transfer, motion, B0 inhomogeneity and multi-element receive, were implemented in this study.

Last, to demonstrate the relationship between the simulation size, the computer configuration and the kernel execution time, several test cases were examined with different combinations of the total pulse sequence time-steps, the total anatomical model isochromats, and the total number and model of GPU devices. The current work demonstrated that a single-GPU computer configuration performed fast simulations of large pulse sequences on a single slice of the anatomical model, whereas a multi-GPU computer equipped with a newer generation of GPU cards performed similarly well on the entire volume of the anatomical model. Although the recorded kernel execution times are highly dependent on the simulation size (size of pulse sequence and anatomical model), this study showed that a multi-GPU computer on the cloud equipped with a newer generation of GPU cards could significantly mitigate the prolonged execution times that accompany more realistic MRI and qMR simulations. Moreover, the latest and future advancements in GPU technology suggest that the performance of the coreMRI simulation platform has the potential to be further improved in the upcoming years.

Despite the latest advancements in GPU and cloud computing and the user-friendliness of the coreMRI simulation platform, the proper design of a simulation experiment still requires the user to have experience in MR physics in order to avoid the appearance of non-realistic artifacts in the simulated MR images. Previous work [12, 13, 37, 38] has already demonstrated that an insufficient frequency spacing among neighbouring isochromats within the anatomical model in combination with a strong-gradients pulse sequence may cause an image quality degradation. The user should be able to properly design the anatomical model based on the design of the pulse sequence and on the question at hand. For this purpose, the coreMRI simulation platform gives the user the ability to use artificial objects in the pulse sequence design, such as software crushers [12].

The work presented here is the first step towards the development of a multi-purpose simulation platform as a web service which is publicly available and common across research groups. The current design of the coreMRI simulation platform, that combines not only high availability, scalability and performance but also compatibility with other platforms and standards (such as the pulseq framework and the ISMRMRD format), is expected to lay the foundations for establishing a simulation standard within the MR field and an online community for developing and sharing pulse sequences, anatomical models and simulation experiments that will eventually promote the MR research and education. Future work will be focused on the expansion of the MR simulation model in other MR applications (such as diffusion) and on the addition of more features (such as custom-defined motion models).

## 5 Conclusions

coreMRI is the first advanced MR simulation platform delivered as a web service through an on-demand, scalable cloud-based and GPU-based infrastructure. coreMRI allows its users to exploit the highly tuned computer performance of GPUs on MR simulations with neither upfront investment for purchasing advanced computer systems nor technical programming expertise. coreMRI is available to the users through the webpage https://www.coreMRI.org. An online and up-to-date documentation is available through the link: https://www.coremri.org/documentation

## Supporting information

S1 Appendix Gradient Echo pulse sequence design with the coreMRI pulse sequence designer. (DOCX)
