# Supplementary material for: coreMRI: A high-performance, publicly available MR simulation platform on the cloud
Source: PLoS One. 2019 May 17;14(5):e0216594. doi: 10.1371/journal.pone.0216594 (PMC6524794; doi:10.1371/journal.pone.0216594)
Supplement: S1 Appendix — (DOCX) [file pone.0216594.s001.docx]

**S1 Appendix. Gradient Echo pulse sequence design with the coreMRI pulse sequence designer.**

For the design of each Gradient Recalled Echo (GRE) pulse sequence, a group of blocks was initially added in the tree-view interface on the left side. This group of blocks held the RF and gradient events that defined a single TR of the pulse sequence and were required for the acquisition of a single k-space line. The RF and gradient pulses within the group of blocks were organized in three separate blocks. The first block held the slice selection process (across the Z axis), the second block held the phase-encoding (PE) process (along the Y axis), the refocusing gradient for the slice selection process (across the Z axis) and the readout prephasor gradient (across the X axis). The third block held the frequency-encoding (FE) process (along the X axis), the acquisition object that defined when the receiver was active and a software crusher at the end of the TR to null the magnetization components on the transverse plane.

In the first block, the magnitude of the slice selection (SS) gradient was expressed as a function of the bandwidth of the RF pulse based on

$${Magnitude}_{G_{SS}}=\frac{{Bandwidth}_{RF}}{gamma*Slice thickness}$$

where the *Bandwidth_RF_* was calculated automatically by the pulse sequence designer during the introduction of the RF pulse, whereas the *gamma* and *Slice thickness* parameters were defined earlier as global parameters of the pulse sequence. In a similar way, the duration of the plateau of the slice selection gradient was set equal to the duration of the RF pulse.

In the second block, the PE order of the GRE pulse sequence was selected to be sequential. For N phase-encoding steps acquired sequentially, the top phase-encoding line of k-space was given by:

$$k_{y_{max}}=\frac{1}{2}\left( N-1 \right)\Delta k_{y}$$

where *N* was the global parameter that defined the size of the k-space in the PE direction and *Δk_y_* was the phase-encoding step size when moving from one TR to the next TR. *Δk_y_* was given by:

$$\Delta k_{y}=\frac{1}{{FOV}_{y}}$$

where *FOV_y_* was the global parameter that defined the size of the FOV in the PE direction. The phase-encoding k-space locations were given by:

$$k_{y}\left( m \right)=\frac{\left( \frac{N-1}{2}-m+1 \right)}{{FOV}_{y}}$$

where *m* was the increasing unique ID of the group of blocks and corresponded to the number of the k-space line. The duration of the PE gradient was equal to

$${Duration}_{G_{PE}}=\frac{\frac{k_{y}\left( 1 \right)}{gamma}}{G_{magn}\left( max \right)}$$

where the numerator defined the area covered by the maximal PE gradient, and the denominator was the global parameter that defined the maximum gradient strength.

In the same block, the duration of the refocusing gradient was set equal to the duration of the phase-encoding gradient, whereas its magnitude was given by:

$${Magnitude}_{G_{SS-refoc.}}=-\frac{\frac{{Area}_{G_{SS}}}{2}}{{Duration}_{G_{SS-refoc.}}}$$

In the third block, the magnitude of the readout gradient was given by:

$${Magnitude}_{G_{readout}}=\frac{{BW}_{receiver}}{gamma*{FOV}_{x}}$$

where *BW_receiver_* was the global parameter that defined the receiver bandwidth and FOV_x_ was the global parameter that defined the size of the FOV in the FE direction. The duration of the readout gradient was given by:

$${Duration}_{G_{readout}}=\frac{{kspace}_{x}}{{BW}_{receiver}}$$

where *k-space_x_* was the global parameter that defined the size of the k-space in the FE direction. Based on the area of the readout gradient, the readout prephasor gradient was added in the second block with its duration being equal to the duration of the phase encoding gradient, whereas its magnitude was given by:

$${Magnitude}_{G_{readout-pref.}}=-\frac{\frac{{Area}_{G_{readout}}}{2}}{{Duration}_{G_{readout-pref.}}}$$

Two more objects were added in the third block: an acquisition object and a software crusher. The duration of the acquisition object was set equal to the duration of the readout gradient, whereas, for reconstruction purposes, a k-space line was assigned to the acquisition object equal to the unique increasing ID of the group of blocks. Next, a software crusher was placed at the end of the group of block (at 10 ms).

The first group of blocks was copied N times equal to the number of k-space lines. The functional programming that was applied during the design of the first group of blocks allowed the automated calculation of the objects’ attributes in the subsequent group of blocks. Last, an ISMRMRD header was formulated through the Pulse Sequence Designer for every pulse sequence to facilitate later the reconstruction of the simulated MR data on the Gadgetron reconstruction framework. In this study, the gradient pulses were modelled with steep ramps.
